# Heat increment of feeding in the common bottlenose dolphin (*Tursiops truncatus*) contributes moderately to field metabolic rate estimates

**DOI:** 10.1242/jeb.251474

**Published:** 2025-12-12

**Authors:** Ioulia Koliopoulou, Stacy L. DeRuiter, Jordi Altimiras, Josefin Larsson, Julietta Arenarez, David Rosen, Andreas Fahlman

**Affiliations:** ^1^ IFM, Linköping University, 581 83 Linköping, Sweden; ^2^ Department of Mathematics and Statistics, Calvin University, Grand Rapids, MI 49546, USA; ^3^ Kolmården Wildlife Park, 618 92 Kolmården, Sweden; ^4^ Fundación Oceanogràfic, 46013 València, Spain; ^5^ Institute for the Oceans and Fisheries, University of British Columbia, Vancouver, BC, Canada, V6T 1Z4; ^6^ Global Diving Research SL, 46004 Valencia, Spain

**Keywords:** Total energy expenditure, Bioenergetic models, Digestion, Marine mammals, Postprandial metabolic rate, Spirometry, Specific dynamic action, Gas exchange, Basal metabolic rate

## Abstract

Digestion elevates metabolism through the heat increment of feeding (HIF) – the energy expended on mechanical and biochemical processes after eating. Quantifying this cost is essential for bioenergetic models that predict energy flow and prey requirements in populations. Using breath-by-breath respirometry, we measured oxygen consumption (*V̇*_O_2__) in eight common bottlenose dolphins (*Tursiops truncatus*) before and after feeding standardized meals (1659–2658 kcal of capelin and herring). Metabolic rate rose by ∼37% above resting levels, peaking 60 min after feeding before returning to baseline within 2 h. When scaled across the day, digestion increased daily metabolic needs by ∼8.2% of basal metabolism, similar to values reported for Steller sea lions (*Eumetopias jubatus*) and harbour seals (*Phoca vitulina*), where HIF contributes 4–10% of daily energy expenditure. This study provides the first multi-individual estimate of HIF in dolphins and suggests that the energetic cost of digestion is a moderate contribution to overall daily metabolism, refining energetic models and improving prey requirement estimates for cetaceans in the wild.

## Teaser

**Summary:** Estimation of the heat increment of feeding in cetaceans shows digestion raises daily metabolism by ∼8.2% of basal metabolic rate in dolphins, a moderate contribution to their energy budgets, refining bioenergetic models and prey requirement estimates.

## INTRODUCTION

Animals must balance energy intake and expenditure by allocating nutritional resources toward somatic maintenance, growth, reproduction and physical activity to optimize fitness (Gadgil and Bossert, 1970). Unexpected changes in either the rate of expenditure or the ability to acquire sufficient prey can have critical impacts at both the individual and population level. There are increasing concerns about the impact of human activities on the behaviour of multiple species of marine mammals (Chudzińska et al., 2024; Wells and Fahlman, 2024). Anthropogenic disturbances can drive behavioural changes in marine mammals, potentially having long-term effects on their ecology, reproductive capacity, body condition and ability to survive (Keen et al., 2021). Concurrently, climate-driven changes in the distribution, structure and abundance of prey may reduce foraging efficiency and negatively affect nutritional status (Learmonth et al., 2006; Rosen and Trites, 2005; Wells and Fahlman, 2024).

An individual's minimal energetic needs that must be met through prey consumption are determined by several essential costs, including basal metabolism, locomotion/foraging costs, thermoregulation and the costs to digest food (Allen et al., 2022; Favilla and Costa, 2020; He et al., 2023; Rosen et al., 2007). Bioenergetic models combine biotic and abiotic influences and predict the mutual relationship between energy expenditure and energy intake (Booth et al., 2023), and are important in predicting how energy acquisition is impacted by anthropogenic stressors. The primary output of bioenergetic models estimates the field metabolic rate (FMR), also called total energy expenditure (TEE), of an animal for different activities, such as foraging, migration, etc., in a specific set of circumstances (Pirotta, 2022). However, what is often of primary concern is the total food required to sustain these activities. To convert FMR into prey intake requires knowledge of prey quality, but also the cost of digestion. Ingested energy from food is ‘lost’ through several processes: fecal energy loss (FEL), urinary energy loss (UEL), and the heat increment of feeding (HIF). HIF refers to the increase in metabolic rate that occurs after the ingestion of a meal (Kleiber, 1961). Although FEL and UEL can be reasonably estimated from the chemical composition of prey, HIF is typically the largest avenue of digestive loss and is highly variable even between closely related species (Secor, 2009).

HIF, also called specific dynamic action (SDA), is the result of mechanical and biochemical processes that accompany food ingestion, including the digestion, absorption and assimilation of a meal (Blaxter, 1989; McCue, 2006; Secor, 2009). The duration and magnitude of HIF depends on several factors, such as the size and energetic content of the meal, the temperature difference between the animal and its prey, the ambient temperature, as well as the age, body size, body composition and overall nutritional state of the animal (Secor, 2009; Wilson and Culik, 1991). For this reason, estimating HIF is not straightforward; it cannot be calculated solely from the composition of the animal's diet, but requires direct comparison of the postabsorptive (the period after digestion and absorption are complete – fasting, catabolic) and postprandial (the period following a meal when the body is processing and absorbing the nutrients – fed, anabolic) metabolic rates (Rosen and Trites, 1997; Wilson and Culik, 1991).

HIF, or at least a component of the postprandial metabolism, has been studied in more than 250 invertebrate and vertebrate species, and a large percentage of those include terrestrial mammals, especially ruminants (Kleiber, 1961; Secor, 2009; Webster, 1983). A number of studies have estimated HIF in marine mammals, which include sea otters (*Enhydra lutris*) (Costa and Kooyman, 1984), harp seals (*Phoca groenlandica*) (Gallivan and Ronald, 1981), harbour seals (*Phoca vitulina*) (Markussen et al., 1994), South American fur seals (*Arctocephalus australis*) (Dassis et al., 2014) and Steller sea lions (*Eumetopias jubatus*) (Rosen and Trites, 1997). While measures of metabolic rate under various conditions have been carried out on bottlenose dolphins (*Tursiops truncatus*) (Fahlman et al., 2018a,b, 2019; Pedersen et al., 2020; Rimbach et al., 2021; van der Hoop et al., 2014; Williams et al., 1993, 2017; Yazdi et al., 1999; Yeates and Houser, 2008), we are only aware of two reports that have attempted to measure HIF in cetaceans: one in the rough-toothed dolphin (*Steno bredanensis*) (Fahlman et al., 2024) and one in the bottlenose dolphin (Yeates and Houser, 2008). The previous study on bottlenose dolphins involved repeated metabolic measurements conducted on separate days and after varying durations following the consumption of 1.4 kg of capelin. This paucity of data is despite the fact that the bottlenose dolphin is widely studied and can be found in managed care worldwide (Hooker and Fahlman, 2024).

In this study, we estimated the course and extent of HIF in bottlenose dolphins through indirect calorimetry, by measuring the oxygen consumption rate (*V̇*_O_2__) and carbon dioxide production rate (*V̇*_CO_2__) before and after the ingestion of a meal. We hypothesized that HIF in the bottlenose dolphin results in a considerable increase in the metabolic rate. However, our results show that it only raises the basal metabolic rate (BMR) over a day by approximately 8.2%. This study is the first to estimate HIF in multiple individuals and indicates that HIF is likely to be a moderate portion of the FMR.

List of abbreviations

BMR: basal metabolic rate
FEL: fecal energy loss
FMR: field metabolic rate
GAMM: generalized additive mixed-effects model
GEI: gross energy intake
HIF: heat increment of feeding
PCoD: population consequences of disturbance
RER: respiratory exchange ratio
RMR: resting metabolic rate
SDA: specific dynamic action
UEL: urinary energy loss
*V̇*_O_2__: oxygen consumption rate
*V̇*_CO_2__: carbon dioxide production rate

## MATERIALS AND METHODS

The experimental procedures in this study were approved by the Animal Care Committee of the Fundación Oceanogràfic (Animal care approval # Oce-32-23).

### Animals

The study was conducted from June to December 2023 with seven adult (mean±s.d. 27±15 years old) and one juvenile (Tt7 in Table 1) bottlenose dolphins (*Tursiops truncatus* Montagu 1821) housed in managed care at Kolmården Wildlife Park, Sweden and Oceanogràfic Aquarium, Spain. The anonymous identification, year of birth, sex and body mass of each individual animal are summarized in Table 1. The mean (±s.d.) body mass and age for the animals participating in the study are reported in Table 2 (although the year of birth was only an estimate for all but 2 individuals, Table 1). The experiments were conducted using operant conditioning. The animals were not restrained and could withdraw from a research trial at any time. Before the start of the study, the dolphins were desensitized to the equipment and trained for the specific research-associated behaviors. Their regular diet consisted of thawed capelin (*Mallotus villosus*) and herring (*Clupea* sp.), and the total daily energetic intake was adjusted for each individual's dietary requirements, based on their body mass, body condition and behavior.

**Table 1.** Summary details of each individual dolphin participating in the study

Animal ID	Sex	Age (years)	Body mass (kg)	Energy intake (kcal)	% Total	Location
Tt1	M	38*	185.6±0.3	2658	35	O
Tt2	M	9	188.3±0.8	2658	16	O
Tt3	M	10	177.9±1.4	1798/2658	18/24	O
Tt4	M	17	172.0±3.4	1798/2658	24/37	O
Tt5	F	38	200.6±0.9	2658	25	O
Tt6	F	40	156.6±0.5	1798	20	K
Tt7	M	5	149.5±3.8	1659	18	K
Tt8	M	39	240.9	2221	19	K
Mean±s.d.		24.5±15.6	183.9±28.4	2258±295	23±6	

Anonymous ID, sex (M/F), age (asterisk represents the estimated age if birthdate is unknown), mean (±s.d.) body mass, and energy intake after measurement of basal metabolic rate in kcal (energy intake) or as a percentage of total daily intake (% total) are shown for each dolphin. Location refers to the managed care facility where the dolphin was housed: either Kolmården Wildlife Park (K) or the Oceanogràfic Aquarium (O). Tt7 was immature.

**Table 2.** Mean (±s.d.) basal metabolic rate (BMR), Kleiber ratio, maximum increase in metabolism due to digestion (Max. ΔMR), specific dynamic action (SDA) scope and postabsorptive respiratory exchange ratio (RER)

ID	BMR (l O_2_ min^−1^)	Kleiber ratio	Max. ΔMR (l O_2_ min^−1^)	SDA scope	RER
Tt1	0.68±0.04 (2)	1.45±0.09	0.31±0.26	1.44±0.35	1.00±0.05
Tt2	0.70±0.24 (4)	1.49±0.50	0.69±0.45	2.24±1.01	0.71±0.04
Tt3	0.95±0.21 (8)	2.10±0.46	0.40±0.16	1.46±0.24	0.76±0.04
Tt4	0.75±0.18 (7)	1.69±0.41	0.17±0.14	1.25±0.20	0.86±0.09
Tt5	0.57±0.23 (4)	1.16±0.47	0.23±0.21	1.50±0.40	0.80±0.10
Tt6	0.73±0.12 (9)	1.77±0.28	0.03±0.26	1.09±0.35	0.83±0.10
Tt7*	0.54±0.12 (7)	1.37±0.30	0.07±0.09	1.14±0.15	0.75±0.04
Tt8	0.89±0.18 (8)	1.41±0.26	0.22±0.13	1.28±0.15	0.75±0.04
Mean±s.d.	0.73±0.14 (49)	1.56±0.29	0.26±0.21	1.42±0.36	0.81±0.09

For BMR, numbers in parentheses are the number of trials. Kleiber ratio was calculated as fasted *V̇*_O_2__/BMR (BMR=0.0093*M*_b_^0.75^, where *M*_b_ is body mass). SDA scope was calculated by dividing peak metabolic rate by BMR (McCue, 2006). *Immature dolphin; and metabolic measurement was considered to be resting metabolic rate (RMR).

### Measuring respiratory flow, gas composition and metabolic rate

The metabolic rate of each individual dolphin was measured using breath-by-breath respirometry, as previously detailed (Fahlman et al., 2015), with additional methodological refinements that were validated against conventional flow-through respirometry in a later study (Allen et al., 2022).

A Fleisch type spirometer, consisting of a laminar flow matrix (Item# Z9A887-2, Merriam Process Technologies, Cleveland, OH, USA), placed in a custom-made housing (ADM+, Valencia, Spain), was placed over the blowhole of the dolphin. The respiratory flow resulted in changes in the differential pressure across the flow matrix, which was measured using a differential pressure transducer (MPX-2.5 mbar type 339/2, Harvard Apparatus, Holliston, MA, USA). Two firm-walled, flexible tubes, each 400 cm in length and with an inner diameter of 1.5 mm, were connected at one end to the spirometer, above and below the flow cell, respectively, and the other end to the pressure transducer to measure pressure deflections associated with breathing (Fahlman et al., 2015). The spirometer was calibrated using a 7.0 l calibration syringe (Series 4900, Hans-Rudolph Inc., Shawnee, KS, USA) immediately before and after each trial, through a series of pump cycles at various flow speeds (Fahlman et al., 2015).

The exhaled gas was sub-sampled through a port in the spirometer, which was connected via a 430 cm long, gas impermeable tube to a fast-response O_2_ and CO_2_ analyser (ML206, Harvard Apparatus). The gas in the spirometer was sampled at a flow rate of 200 ml min^−1^, with a response time for a 90% change to equilibrium for O_2_ and CO_2_ of 67 ms and 94 ms, respectively (Fahlman et al., 2015). A mathematical correction for the response time was used as detailed in Allen et al. (2022). The gas analysers were connected to a data acquisition system (Powerlab 8/35, ADInstruments, Colorado Springs, CO, USA), sampled and recorded at 2000 Hz and displayed on a laptop computer running LabChart (v7.3.7, ADInstruments). The respiratory gas signals were phase-corrected to match the respirations, to account for the lag resulting from gas flow through the tubing.

The expiratory flow and expired O_2_ and CO_2_ concentrations were used to calculate the instantaneous *V̇*_O_2__ (l O_2_ min^−1^) and *V̇*_CO_2__ (l CO_2_ min^−1^), as previously detailed (Allen et al., 2022; Fahlman et al., 2015). The instantaneous *V̇*_O_2__ and *V̇*_CO_2__ were integrated to yield the total volume of O_2_ and CO_2_ exchanged for each breath. The volumes were summed for each breath during the trial period and divided by the duration of the trial to provide an estimate of the *V̇*_O_2__ and *V̇*_CO_2__ for that period. The duration of metabolic rate (*V̇*_O_2_ _and *V̇*_CO_2__) measurements varied between 5 and 7 min. In addition, the respiratory flow was also used to calculate the tidal volume for each breath and the duration between breaths was used to calculate the breathing frequency.

The gas analyser was calibrated before the experiment using a commercial mixture of 4% O_2_, 5% CO_2_ and balance argon (Arcal, Airliquide). Ambient air was used to check the calibration before and after each experimental trial. To make appropriate respirometry corrections (see ‘Data analysis’, below) the daily air temperature and humidity were recorded using a digital thermometer and hygrometer and were on average 21.6±4.4°C (range: 4.3–35.6°C) and 55±16% (range: 21–100%), respectively. The average water temperature during trials was 22.7±1.3°C (range: 20.5–23.7°C) and 23.6±0.09°C (range: 23.5–23.7°C), at the Oceanogràfic and Kolmården Wildlife Park, respectively.

### Temporal metabolic response of digestion

Each research trial consisted of one postabsorptive (pre-fed/fasted) and one to three postprandial measurements (post-fed). Typically, HIF causes metabolism to increase significantly to a peak over time, and then more slowly decrease back to baseline (fasted) level (Rosen and Trites, 1997). For this reason, it would have been ideal to measure the metabolic rate continuously throughout the course of the trial to capture the full temporal dynamics of HIF. However, this was not possible given the constraints of the study design. Instead, we used serial subsampling at the different time points along the course of the HIF effect and then used these data to model the average temporal response in metabolism (see ‘Statistical analysis’, below). This, in turn, allowed us to estimate the overall increase in oxygen consumption over resting levels, and express this relative to ingested energy.

The fasted measurement was performed following an overnight fast with the dolphin inactive while positioned at the side of the pool and breathing spontaneously through the pneumotachometer. This allowed the measurement of respiratory flow and expired gas composition. The time of the last meal on the day prior to the trial was recorded and used to determine the fasting duration for each animal. Next, the dolphin was given a predetermined quantity of food, a 1–1.5 kg mixture of capelin (12.6% fat, 13.4% protein, 70.4% water, energy density 1659 kcal kg^−1^) and herring (8.1% fat, 16.9% protein, 72.3% water, energy density 1406 kcal kg^−1^), which corresponded to between 1659 and 2658 kcal (Table 1). Metabolic measurements were then repeated 30, 60, 90 and/or 120 min after the meal, without additional food being given between trials. The dolphins were free to swim between measurements. For some trials, a single post-feeding measurement was taken, whereas for others, up to three repeated measurements were made without the provision of food in-between. For all individuals, a minimum of three trials per caloric intake were obtained. For two individuals, two different caloric intake levels were tested, 2658 kcal and 1798 kcal (Tt2 and Tt3; Table 1).

### Data analysis

All gas volumes were converted to standard temperature and pressure dry (STPD; Quanjer et al., 1993), as previously detailed (Fahlman et al., 2015). Exhaled air was assumed saturated at 37°C, and inhaled air volume was corrected for ambient temperature and relative humidity. First, the mean BMR was calculated from the *V̇*_O_2__ of the fasted trials. These measures met standardized criteria for BMR: dolphins were adult, postabsorptive, quiescent and within their thermoneutral zone (Kleiber, 1961), except for one animal that was a juvenile (Tt7; Table 1). Thus, as these measurements were done under basal conditions in all except dolphin Tt7, we refer to these as BMR. The measurements that followed were not considered postabsorptive and are referred to as resting metabolic rate (RMR). In other words, BMR before feeding and RMR at 30, 60, 90 and 120 min following a meal. Oxygen volumes were converted to energy equivalents using an energetic coefficient of 4.8 kcal l^−1^ O_2_, consistent with previous marine mammal studies (McCue, 2006).

For measures of BMR from the fasted trials, the respiratory exchange ratio (RER) was calculated as *V̇*_CO_2__/*V̇*_O_2__. The Kleiber ratio was calculated as the fasted *V̇*_O_2__ measurement divided by the estimated BMR from the equation for terrestrial mammals introduced by Kleiber (1961) (BMR=0.0093*M*_b_^0.75^, where BMR is in l O_2_ min^−1^ and *M*_b_ is body mass in kg; Table 2).

For the feeding trials, the change in metabolism after feeding was calculated as the difference between the recorded metabolic rate for each time point and the BMR. HIF was computed as the absolute increase in energy consumption above BMR, expressed as a percentage of gross energy intake (GEI). The specific dynamic action (SDA) scope represented the relative increase in metabolism, and was calculated by dividing the peak metabolic rate by the BMR (McCue, 2006) (Table 3).

**Table 3.** SDA scope for different species of marine mammals

Common name	Genus/species	SDA scope	Reference
South American fur seal	*Arctocephalus australis*	1.6	Dassis et al., 2014
Northern fur seal	*Callorhinus ursinus*	1.6	Liwanag, 2010
Steller sea lion	*Eumetopias jubatus*	1.7–2.1	Rosen and Trites, 1997
Harp seal	*Phoca groenlandica*	1.4–1.7	Gallivan and Ronald, 1981
Harbour seal	*Phoca vitulina*	1.1–1.7	Markussen et al., 1994
Sea otter	*Enhydra lutris*	1.5	Costa and Kooyman, 1984
Rough-toothed dolphin	*Steno bredanensis*	0.9–1.7	Fahlman et al., 2024
Bottlenose dolphin	*Tursiops truncatus*	1.6	Yeates and Houser, 2008
Bottlenose dolphin	*Tursiops truncatus*	1.3–1.6	This study

### Statistical analysis

Statistical analysis was performed using R version 4.4.3 (http://www.R-project.org/) in the RStudio integrated development environment (https://posit.co/). The variation for all mean values is reported as ±1 standard deviation (s.d.), unless otherwise specified. Paired *t*-tests were used to compare mean values.

A normal generalized additive mixed-effects model (GAMM) was used to investigate the relationship between *V̇*_O_2_ _and various predictor variables. We fitted the model with the *gam* function from the R package *mgcv* (mgcv package, v1.8-34; Wood, 2011), using maximum likelihood estimation, and allowing for the addition of an extra penalty term that potentially allows each smooth term to be penalized to zero to avoid overfitting (via the *select* input to *gam*). The variables time, energy intake, age, body mass and pool temperature were included as smooth terms (with thin plate regression spline bases with shrinkage and specifying a basis dimension of 4 for each smooth), representing potentially non-linear relationships. We allowed for an interaction between time and energy intake (via a tensor product smooth term, again with a basis dimension 4). We also included sex as a binary predictor and included random effects of animal ID and day of trial to accommodate potential differences between individuals and days. Model conditions related to residual normality, independence and the mean–variance relationship were verified using graphical checks (see Supplemental Materials and Methods and Figs S2, S3 and S4). Type II ANOVA (*anova* function in R) was used to test the null hypothesis of no association between *V̇*_O_2_ _and each predictor variable, while standard deviation estimates for random intercept terms were obtained using the *mgcv* package function *gam.vcomp*.

To facilitate comparison of model results with observed data, we computed a time course of expected *V̇*_O_2_ _values according to the GAMM at the population level (excluding random-effect terms), and for marginal mean values of all other predictors. To enable estimation of standard errors and percentile-based interval estimates for GAMM predictions and derived quantities, we used a resampling approach, repeating the calculation for 10,000 samples of model parameter values, obtained via Metropolis–Hastings sampling from the posterior (using the *gam.mh* function from the *mgcv* package). To estimate the total volume of O_2_ consumed over the full 133 min trial period, we computed the area under the curve, representing the average results for all animals, of the GAMM predictions via trapezoidal integration (*cumtrapz* function from the *pracma* R package; https://CRAN.R-project.org/package=pracma).

All data and code used to carry out analysis and produce figures are provided in the Supplemental Materials and Methods; also see https://doi.org/10.5281/zenodo.16804429.

## RESULTS

### Metabolic rate, breathing frequency and tidal volume

The average mass-specific *V̇*_O_2__ (ml O_2_ min^−1^ kg^−1^) under BMR conditions was 4.0±0.8 ml O_2_ min^−1^ kg^−1^. The postabsorptive RER was within normal range for all individuals, i.e. all values ranged between 0.7 and 1.0 (Table 2). There were no differences in the average postabsorptive or postprandial (0.80±0.08) RER (*t*=0.472, *P*=0.651). The average tidal volume and breathing frequency under BMR conditions were, respectively, 3.7±1.3 l and 2.8±1.3 breaths min^−1^.

### Temporal effect of digestion on metabolism and lung function

#### Metabolism

**Fig. 1.**
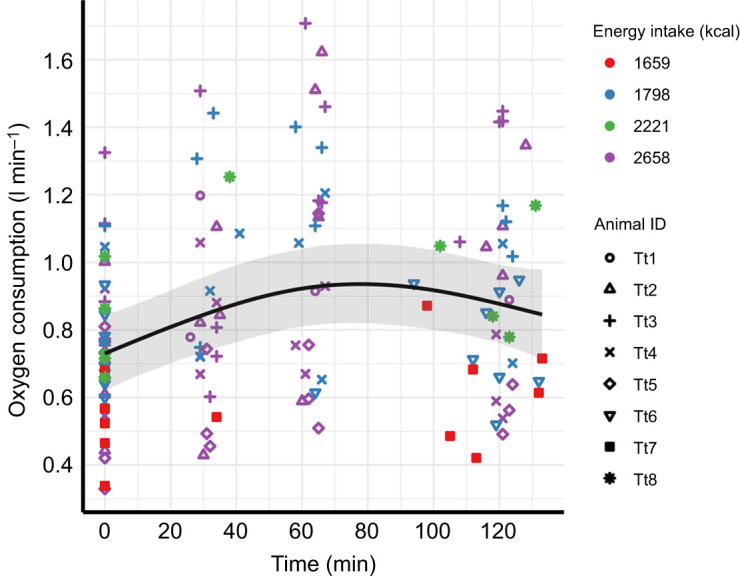
**Temporal response in metabolism following ingestion of a meal.** The metabolic rate (*V̇*_O_2__) at time 0 is the basal metabolic rate (BMR) measured before meal ingestion. The different individual dolphins are indicated by different symbols (Table 1) and variation in caloric intake is indicated by colour. The solid black line is the expected oxygen consumption according to the GAMM, with the shaded region indicating the 95% confidence interval. The GAMM analysis indicated an increase in metabolic rate during the first 60 min following ingestion of a meal, followed by a slow decline that was approximately 10% above basal levels at 133 min (*F*=9.1, *P*=5.4×10^−5^; see Supplementary Materials and Methods for additional details).

The average maximal increase in *V̇*_O_2_ _(max. MR in Table 2) was 36% (from 0.73 to 0.99 l O_2_ min^−1^) following an average energy intake that represented 23% of the daily total (Tables 1 and 2, Fig. 1). There was considerable variation in the response, but there was a general trend for an increase that was observed after 30 min, with an estimated peak time about 60 min after food ingestion (Fig. 1; see Fig. S1 for individual variation). Following this, the RMR decreased towards BMR at both 90 and 133 min (the longest duration of any trial). This postprandial pattern was further supported by the GAMM, which showed very strong evidence of an association between time after feeding and *V̇*_O_2__ (*F*=9.1, *P*=5.4×10^−5^; Supplemental Materials and Methods), indicating a clear non-linear response (Fig. 1). The model also revealed substantial variation between individuals and trial days (with estimated random effect standard deviations of 0.13 and 0.087, comparable in magnitude to the residual standard deviation of 0.20). In contrast, there was no evidence of an interaction between energy intake and time after feeding, or of *V̇*_O_2__ being associated with energy intake, age, body mass, pool temperature or sex (all *P*>0.05; Supplemental Materials and Methods, Figs S1–S4, Table S1).

#### The metabolic cost of digestion

The SDA scope is summarized in Table 2. The mean (±s.e.m.) area under the curve from the GAMM predicted that the volume of O_2_ consumed during the 133 min trial period was 116.7±7.1 l O_2_ (Fig. 1; Figs S5–S7, Tables S2–S4). (These calculations were for the marginal mean values of predictors other than time since feeding, namely: fed 23% of daily total food intake, body mass 179.4 kg, age 21.4 years, pool temperature 22.8°C and 76% male.) Without HIF, the same value for BMR during a 133 min period would be 97.1±7.6 l (i.e. 0.73±0.06 l O_2_ min^−1^×133 min). Thus, the metabolic cost to digest 23% of the daily food consumed was 19.6 l O_2_, or an increase of 20% above BMR. To be able to estimate the total daily metabolic contribution of digestion, we assumed that the HIF response is linearly related to the caloric intake. Although we did not find a relationship with caloric intake, such a relationship has been reported in other studies (Fu et al., 2005; Secor, 2009). Based on this assumption, the total metabolic cost of digestion for a day would be 85.7±19.2 l O_2_, whereas BMR over 24 h would consume 1051.3±81.8 l O_2_. Thus, the measured digestive cost was 8.2±1.8% of the daily BMR. Based on the results from the GAMM, the HIF of the 8 dolphins was calculated as the excess O_2_ consumed over the 133 min (19.6 l O_2_), converted to kcal, divided by the average energy intake of the animals (Table 1). This resulted in a value for HIF as 4.2% GEI.

## DISCUSSION

In the present study, the BMR, RMR and HIF were measured before (BMR) and at 30, 60, 90 and 120 min (RMR) after the ingestion of a meal. The mean BMR was 4.0±0.8 ml O_2_ min^−1^ kg^−1^, which was an average of 1.56×Kleiber's prediction for BMR in terrestrial mammals (Table 2) but similar to that reported for bottlenose dolphins and smaller marine mammals (Allen et al., 2022; Dassis et al., 2014; Fahlman et al., 2024; He et al., 2023; Yazdi et al., 1999; Yeates and Houser, 2008). Similar to other studies measuring the digestive costs in marine mammals (Fahlman et al., 2024; Rosen and Trites, 1997; Yeates and Houser, 2008), there was an initial increase in metabolic rate, followed by a decrease towards postprandial levels. The maximal increase in RMR was observed 60 min following feeding and then approached pre-feeding values but was still about 10% higher at around 120 min following feeding (Fig. 1). The mean peak RMR (5.5±1.4 ml O_2_ min^−1^ kg^−1^) following feeding was 35% above BMR (4.0±0.8 ml O_2_ min^−1^ kg^−1^). In addition, the temporal response and the magnitude of the metabolic cost of HIF were similar to those reported from a single measurement in the same species and the result presented in the rough toothed dolphin (*S. bredanensis*) (Fahlman et al., 2024; Yeates and Houser, 2008).

### Limitations and future perspectives

It is known that the metabolic effects of digestion, i.e. HIF, show a temporal response in which the metabolic rate initially increases and then decreases back to the postabsorptive levels (McCue, 2006; Rosen and Trites, 1997; Secor, 2009). Thus, to accurately describe this time course, it is important to obtain enough samples at different times. In a previous study, the authors estimated the temporal response by repeating the RMR measurements on different days (Yeates and Houser, 2008). In the present study, we wanted to account for the effect of daily variation and measured the RMR at different time points following the BMR measurement. For this reason, we were limited by each individual's behavior and willingness to participate in each repeated measurement, making it particularly challenging during later measurements for which no food was given. This limited the number of repeated measurements that could be performed during a trial. As a result, a control trial in which no food was given between the first and later measurements was not possible. In addition, both the type of food and its caloric content alter the magnitude and temporal response of HIF (Beamish, 1974; Tandler and Beamish, 1980). In this study, we therefore limited the scope to a narrow range of food types and a restricted caloric intake, reducing dietary variation and better isolating the metabolic effect of digestion. Nevertheless, other sources of variability, such as physiological differences, daily behavioral fluctuations and limited repeated measurements per animal, still introduced significant noise in the dataset, making it difficult to detect clear patterns or effects. Despite these limitations, we were able to obtain data from eight bottlenose dolphins and provide an estimate of the magnitude of the metabolic effects associated with digestion, along with the contribution of HIF to the daily FMR.

### Heat increment of feeding

The 8 individuals were given a diet of 1–1.5 kg of capelin and herring, corresponding to 1659–2658 kcal (mean: 2285±388 kcal), or 18–37% of the total calories consumed in a day (mean: 23±7%; Table 1). This caloric intake increased the mean metabolic rate by 35% (37% including the non-adult animal Tt7) above the measured BMR. It is known that meal size affects both the magnitude and temporal dynamics of HIF (Secor, 2009). Given the narrow range of meal types and sizes, it was not surprising that the variation in caloric intake did not affect the results in the current study. In fact, the meal sizes may have different effects on HIF depending on the species studied, and larger differences in caloric intake may be necessary to elicit detectable changes in metabolic response (Fu et al., 2005; McCue, 2006). For example, in Steller sea lions consuming 4 kg herring, the metabolic rate increased by 12.4±0.9%, compared with 9.9±0.9% after consuming 2 kg (Rosen and Trites, 1997). The findings of the present study are consistent with the results presented by Yeates and Houser (2008), in which the metabolic rate of a 220 kg bottlenose dolphin that consumed 1800 kcal increased by between 20% and 40% from 25 to 100 min following feeding. For the rough-toothed dolphin, the SDA scope varied more, but was within the same range (Fahlman et al., 2024). This larger variation may be due to a mixture of trials where the RMR was measured once, either 60 min or 120 min following feeding. While our GAMM did not identify an association between percentage GEI consumed and postprandial *V̇*_O_2__, we report HIF as a percentage of GEI to maintain consistency with other studies and to enable integration with bioenergetic models. This standardized metric reflects the relative metabolic cost of digestion, facilitating comparisons across individuals, caloric intake or species, and supports the use of percentage HIF in contexts where absolute energetic predictors may show high individual variability or limited statistical power.

Using the results from the current study, it is possible to provide an estimate of the total daily digestive metabolic cost. Such information is important to better inform bioenergetic models and make more accurate predictions to evaluate the potential impact of disturbance, for example via population consequences of disturbance (PCoD) models (Pirotta et al., 2018; Winship et al., 2002). Using the results from the GAMM, we estimated that for a bottlenose dolphin given 23% of its daily total caloric intake, the metabolic rate for the next 133 min increased by an average 20%. Scaling this up to the full caloric intake for the entire day, we estimated that the metabolic cost for digestion would be 8.2% of BMR (see Results, ‘The metabolic cost of digestion’). As it is possible that the effect of digestion had not finished at 130 min (Fig. 1), the overall contribution of HIF to FMR increase may be a bit higher than estimated here. Extrapolating the curve in Fig. 1 suggests that metabolic rate might have returned to BMR by approximately 170 min. This duration also agrees with the data reported in Yeates and Houser (2008), where the metabolic rate had also returned to basal levels after 170 min. Assuming a RMR of 0.8 l O min^−1^ between 133 and 170 min yields an additional 29.6 l O_2_. If this increase represents 23% of the daily food intake, the total daily increment in oxygen consumption due to digestion would be 128.7 l O_2_, compared with 1051.3 l O_2_ from BMR, equivalent to a 12.2% increase. This estimate probably represents the maximal average elevation in metabolic rate associated with digestion. The SDA scope provides a useful index for comparing the relative magnitude of HIF between species, and represents the ratio of peak metabolic rate to BMR (McCue, 2006). For the bottlenose dolphin, the SDA scope ranged between 1.3 and 1.6 (Table 3). In comparison, the SDA scope in juvenile otariids was similar to or slightly higher than that of the bottlenose dolphin, at between 1.6 and 2.1 (Dassis et al., 2014; Liwanag, 2010; Rosen and Trites, 1997), whereas in one adult harp seal (*Phoca groenlandica*) and four juvenile harbour seals (*Phoca vitulina*) it was within a similar range, 1.1–1.7 (Gallivan and Ronald, 1981; Markussen et al., 1994). One reason for the higher values in the otariids could be that these were mostly smaller juvenile or weaned animals, where smaller size and limited insulation may result in a greater proportion of thermal substitution from digestion to make up a larger portion of the metabolic cost to keep warm (Costa and Kooyman, 1984). However, a follow up study in the juvenile Steller sea lions did not support that thermal substitution occurred in this species (Rosen and Trites, 2003). Thus, the range of SDA scope in different species may be within the natural variation.

### Conclusion

There is an increasing need to better understand the energetic requirements of marine mammals, particularly considering the potential effect that human disturbances and climate change may have on their survival. Models aimed at predicting how disturbances may alter population levels (PCoD) require a better understanding of the eco-physiology of the study species (Chudzińska et al., 2024; Pirotta et al., 2018). The energetic requirements of a species have been identified to be one of the variables to which the outcome of PCoD models is most sensitive, and it is therefore important to carefully quantify the flow of energy within the organism and between different trophic levels (Chudzińska et al., 2024; Winship et al., 2002). Nevertheless, there is limited knowledge of the physiology of marine mammals and how related constraints affect survival. The data presented in this study provide estimates of the digestive contribution to FMR and suggest that it increases the daily energetic needs of bottlenose dolphins by 8.2% of BMR. These data will help improve estimates from bioenergetics models and contribute to our understanding of how a changing environment may alter survival in this species.

## Supplementary Material

10.1242/jexbio.251474_sup1 Supplementary information
