# Supplementary material for: Heat increment of feeding in the common bottlenose dolphin (Tursiops truncatus) contributes moderately to field metabolic rate estimates
Source: J Exp Biol. 2025 Dec 12;228(24):jeb251474. doi: 10.1242/jeb.251474 (PMC12752490; doi:10.1242/jeb.251474)
Supplement: Supplementary information [file jexbio-228-251474-s1.pdf]

Supplementary Materials and Methods

Table of contents

R Setup 1
Data Preparation 2
Data by Individual 2
HIF Model 5
  Model fitting 5
  Model Checking 6
    Residual Histogram 7
    Scaled Residual Plot 8
    Residual ACF Plot 9
  Model Interpretation 10
    Hypothesis Testing 10
    Variance Term Estimation 11
    Model prediction note 12
    Marginal Means of Model Variables 13
    Predictions with uncertainty 14
References 22

R Setup

```
library(mgcv)
library(ggformula)
```

```
library(tidyverse)
library(readxl)
library(ggeffects)
library(pracma)
library(DHARMa)
library(gt)
library(gtsummary)
# remotes::install_github("m-clark/mixedup")
library(mixedup)

knitr::opts_chunk$set(echo = TRUE,
                      fig.width = 6,
                      fig.height = 4.5,
                      fig.path = "figures/HIF-",
                      dpi = 300,
                      dev = c("CairoJPEG", "cairo_pdf"),
                      warning = FALSE,
                      message = FALSE
                      )

theme_set(theme_minimal(base_size = 12))
```

Data Preparation

```
HIF_data <- read_xlsx('data/updated_data.xlsx',
                      .name_repair = 'unique_quiet') |>
  mutate(animal = factor(animal),
         animal2 = fct_reorder(animal, kcal, min),
         day = factor(interaction(animal, date, drop = TRUE)),
         # numeric version of binary sex variable
         # (this doesn't change model parameter estimates but
         # eases later predictions from fitted model)
         is_male = if_else(sex == "M", 1,0))
```

Data by Individual

```
kcal_colors <- c("#E41A1C", "#377EB8", "#4DAF4A", "#984EA3")
shape_vector <- c("Tt1" = 1, "Tt2" = 2, "Tt3" = 3, "Tt4" = 4,
                  "Tt5" = 5, "Tt6" = 6, "Tt7" = 7, "Tt8" = 8)
```

```
HIF_ave <- HIF_data |>
  group_by(animal2, time, kcal) |>
  summarise(oxygen_cons = mean(oxygen_cons, na.rm = TRUE),
            exact = mean(exact, na.rm = TRUE),
            .groups = "drop")
```


Journal of Experimental Biology • Supplementary information

```r
oxygen_time_ind_plot <-
  ggplot(HIF_data,
         aes(x = exact, y = oxygen_cons)) +
  geom_point(aes(
    color = factor(kcal),
    shape = animal2),
    size = 1,
    # color = "grey64",
    stroke = 1.2,
    alpha = 0.3
    ) +
  scale_shape_manual(values = shape_vector) +
  facet_wrap("animal2", ncol = 2) +
  geom_point(aes(x = exact, y = oxygen_cons, color = factor(kcal)),
             data = HIF_ave) +
  geom_line(aes(x = exact, y = oxygen_cons, color = factor(kcal)),
            data = HIF_ave) +
  labs(x = "Time (min)",
       y = "Oxygen Consumption (L / min)",
       color = "Energy Intake (kcal)",
       shape = "Animal ID") +
  scale_color_manual(values = kcal_colors) +
  theme_minimal() +
  theme(axis.text = element_text(size = 12),
        axis.title = element_text(size = 14),
        axis.line = element_line(linewidth = 1)) +
  scale_x_continuous(breaks = seq(0, 120, by = 20)) +
  scale_y_continuous(breaks = seq(0, 2, by = 0.2))

print(oxygen_time_ind_plot)
```

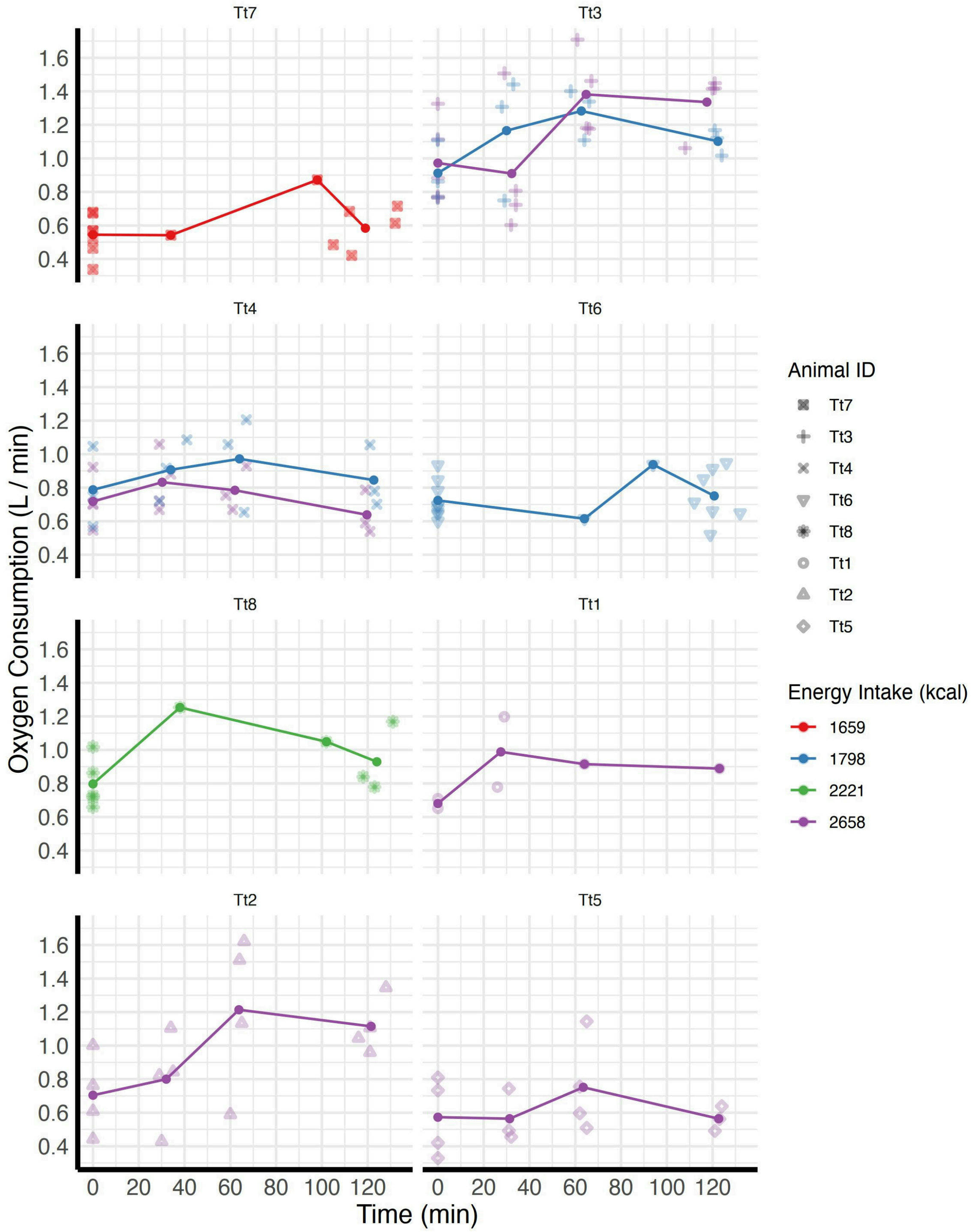

Fig. S1. Oxygen consumption as a function of time, by individual dolphin. Colors indicate energy intake in kcal. At each timepoint, varying symbols for each dolphin plot individual replicate measurements, while dots plot means at each timepoint. Nominal timepoints were 0, 30, 60, and 120 minutes, but observations are plott d at the exact time they were actually taken (or, for means, the average of those exact times).

Journal of Experimental Biology • Supplementary information

HIF Model

Model fitting

```
HIF_model <- gam(oxygen_cons ~
                   # smooth terms
                   s(exact, k = 4, bs = "ts") +
                   s(percentdailytotal, k = 4, bs = "ts") +
                   ti(exact, percentdailytotal, k = 4, bs = "ts") +
                   s(body_mass, k = 4, bs = "ts") +
                   s(age, k = 4, bs = "ts") +
                   # other predictor(s)
                   # treat sex as logical 0/1 for convenience later when
predicting
                   is_male +
                   s(pool_temp, k = 4, bs = "ts") +
                   # random effects
                   s(animal, bs = "re") +
                   s(day, bs = "re"),
                 data = HIF_data,
                 # to use ANOVA for hypothesis testing after model fitting
                 method = "ML",
                 # to avoid overfitting and allow penalization of smooths
                 # to flat lines if needed
                 select = TRUE)
```

Standard model summary output from `gam()`:

```
summary(HIF_model)
```

```

Family: gaussian
Link function: identity

Formula:
oxygen_cons ~ s(exact, k = 4, bs = "ts") + s(percentdailytotal,
    k = 4, bs = "ts") + ti(exact, percentdailytotal, k = 4, bs = "ts") +
    s(body_mass, k = 4, bs = "ts") + s(age, k = 4, bs = "ts") +
    is_male + s(pool_temp, k = 4, bs = "ts") + s(animal, bs = "re") +
    s(day, bs = "re")

Parametric coefficients:
            Estimate Std. Error t value Pr(>|t|)
(Intercept)   0.6920     0.1025   6.751 7.21e-10 ***
is_male       0.1819     0.1189   1.530    0.129
---
Signif. codes:  0 '***' 0.001 '**' 0.01 '*' 0.05 '.' 0.1 ' ' 1
```


Journal of Experimental Biology • Supplementary information

```
Approximate significance of smooth terms:
                                  edf Ref.df      F  p-value    
s(exact)                    2.000e+00      3  9.077 5.42e-05 ***
s(percentdailytotal)        2.463e-01      3  0.797   0.2530    
ti(exact,percentdailytotal) 6.440e-01      9  0.186   0.1127    
s(body_mass)                2.199e-06      3  0.000   0.5413    
s(age)                      5.244e-07      3  0.000   0.6708    
s(pool_temp)                1.572e-06      3  0.000   0.9907    
s(animal)                   4.654e+00      6 14.341 4.10e-07 ***
s(day)                      1.347e+01     46  0.525   0.0223 *  
---
Signif. codes:  0 '***' 0.001 '**' 0.01 '*' 0.05 '.' 0.1 ' ' 1

R-sq.(adj) =  0.531   Deviance explained = 60.9%
-ML = -4.0912  Scale est. = 0.040124  n = 133
```

Below is the model summary table:

```
tbl_regression(HIF_model)
```

**Characteristic**	**Beta**	**95% CI**	**p-value**
is_male	0.18	–0.05, 0.41	0.13
s(exact)			<0.001
s(percentdailytotal)			0.3
ti(exact,percentdailytotal)			0.11
s(body_mass)			0.5
s(age)			0.7
s(pool_temp)			>0.9
s(animal)			<0.001
s(day)			0.022

Abbreviation: CI = Confidence Interval

Table S 1: GAMM Model summary table.

Model Checking

Below are `mgcv` package checks for GAMs, presented mainly to view model convergence and basis dimension checking results.

Journal of Experimental Biology • Supplementary information

```r
gam.check(HIF_model)
```

```

Method: ML   Optimizer: outer newton
full convergence after 19 iterations.
Gradient range [-7.932546e-07,4.625727e-07]
(score -4.091216 & scale 0.04012398).
Hessian positive definite, eigenvalue range [4.039797e-07,67.38306].
Model rank =  82 / 82

Basis dimension (k) checking results. Low p-value (k-index<1) may
indicate that k is too low, especially if edf is close to k'.

                                  k'      edf k-index p-value
s(exact)                    3.00e+00 2.00e+00    1.02    0.57
s(percentdailytotal)        3.00e+00 2.46e-01    0.97    0.32
ti(exact,percentdailytotal) 9.00e+00 6.44e-01    1.02    0.46
s(body_mass)                3.00e+00 2.20e-06    1.00    0.44
s(age)                      3.00e+00 5.24e-07    1.00    0.48
s(pool_temp)                3.00e+00 1.57e-06    0.98    0.35
s(animal)                   8.00e+00 4.65e+00      NA      NA
s(day)                      4.80e+01 1.35e+01      NA      NA
```

Residual Histogram

This graph allows us to verify the residual normality condition.

Journal of Experimental Biology • Supplementary information

```
gf_dhistogram(~resid(HIF_model), bins = 15) |>
  gf_fitdistr(dist = "dnorm") |>
  gf_labs(x = "GAMM Residuals",
          y = "Density")
```

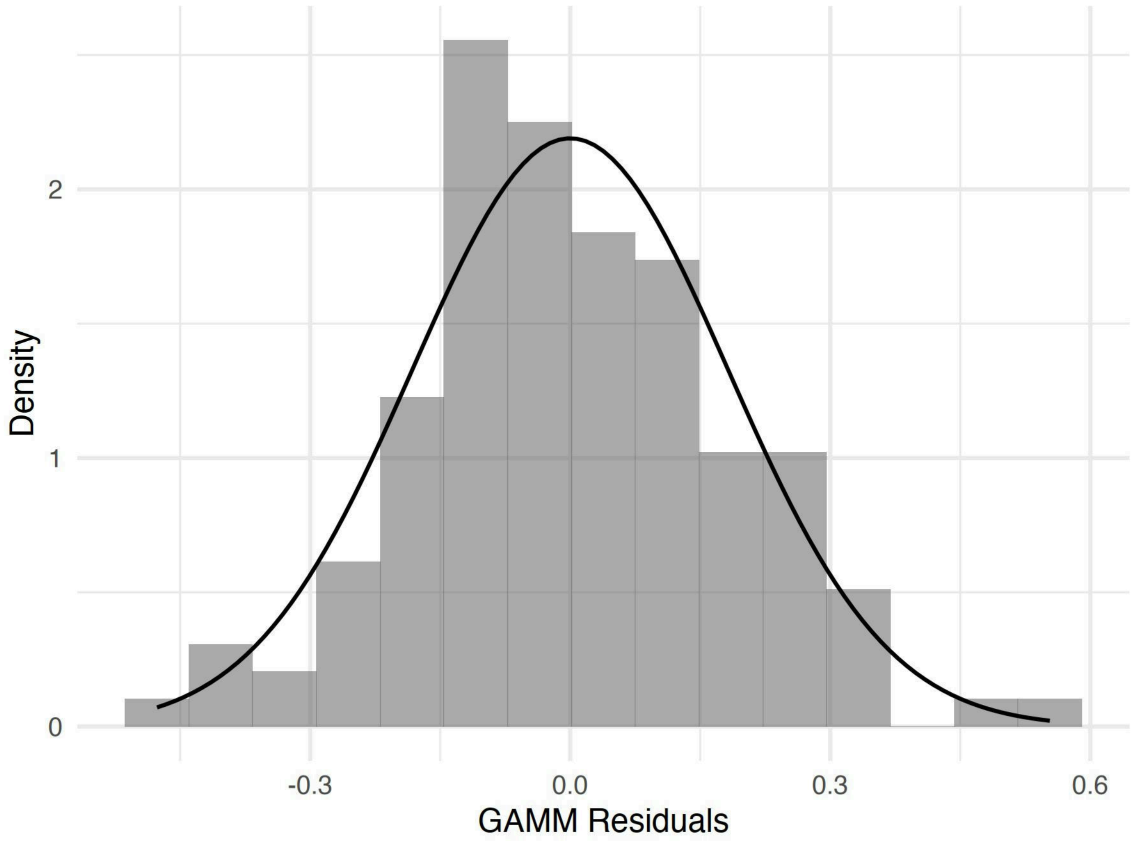

Fig. S2. Residual histogram.

Scaled Residual Plot

The scaled residual plot (Hartig, 2024) allows verification that the mean-variance relationship is as expected, particularly for mixed-effect models and generalized linear models. If all is well, scaled residuals will be trendless and will have uniform spread between 0-1 for all fitted values.

Journal of Experimental Biology • Supplementary information

```
plotResiduals(simulateResiduals(HIF_model), quantreg = FALSE)
```

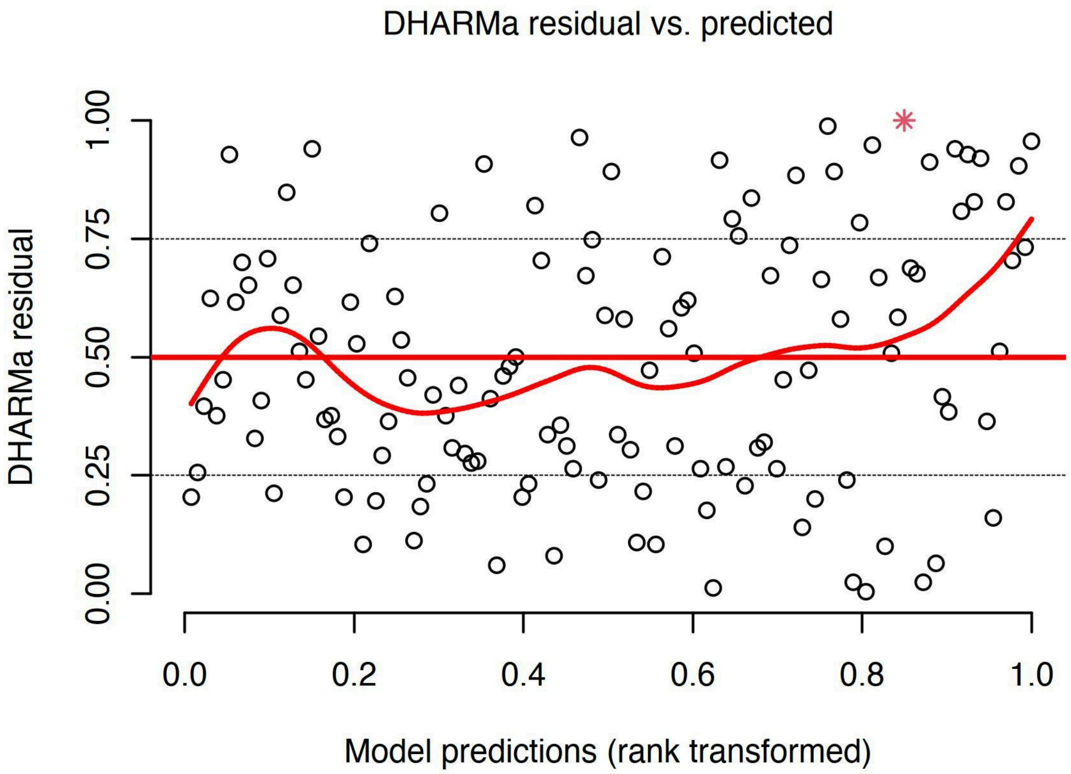

Fig. S3. GAMM scaled residual plot

Residual ACF Plot

The ACF plot allows us to verify the residual independence condition.

Journal of Experimental Biology • Supplementary information

```
acf(resid(HIF_model), main = "")
```

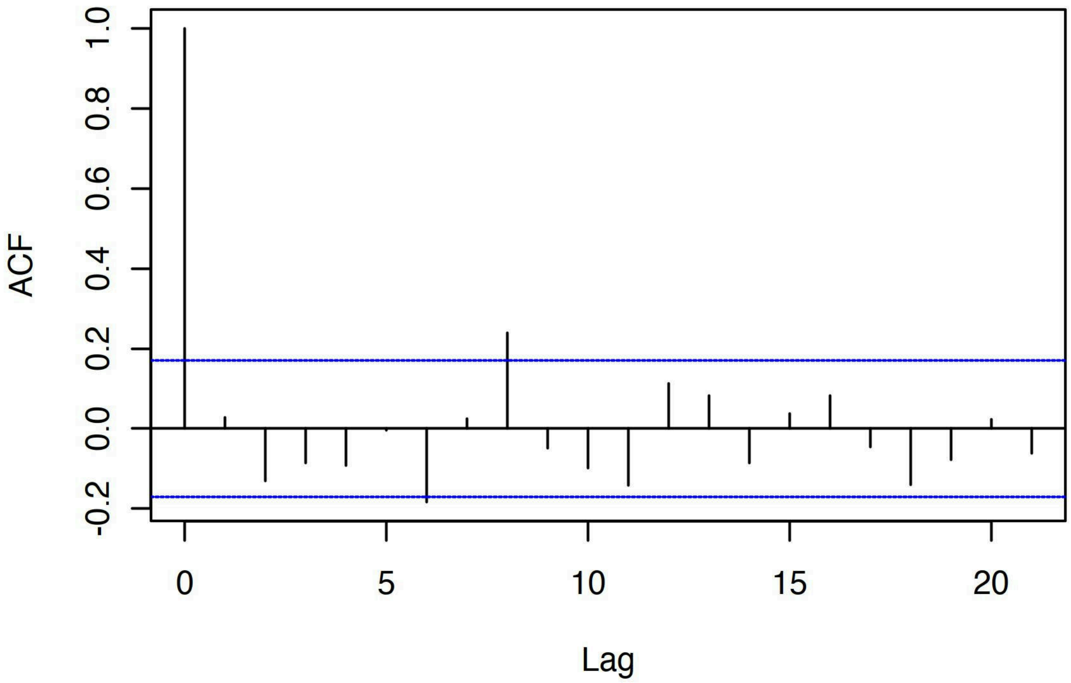

Fig. S4. GAMM Residual ACF plot.

Model Interpretation

Hypothesis Testing

Type II ANOVA allows us to test the null hypothesis of no association with the response, for each predictor (and interaction) included in the model.

Journal of Experimental Biology • Supplementary information

```
my_atab <- anova(HIF_model)$s.table
terms <- rownames(my_atab)
tibble(Term = terms,
       EDF = my_atab[,"edf"],
       `Reference DF` = my_atab[,"Ref.df"],
       F = my_atab[,"F"],
       `p-value` = my_atab[,"p-value"]
       ) |>
  gt()
```

Term	EDF	Reference DF	F	p-value
s(exact)	1.999898e+00	3	9.076647e+00	5.419976e-05
s(percentdai-lytotal)	2.463071e-01	3	7.966751e-01	2.529762e-01
ti(exact,per-centdailytotal)	6.440355e-01	9	1.862620e-01	1.126831e-01
s(body_mass)	2.198842e-06	3	2.780387e-07	5.412923e-01
s(age)	5.244190e-07	3	3.164028e-08	6.707563e-01
s(pool_temp)	1.571967e-06	3	1.155409e-09	9.907114e-01
s(animal)	4.654132e+00	6	1.434134e+01	4.104891e-07
s(day)	1.346616e+01	46	5.252097e-01	2.225639e-02

Table S 2: ANOVA table for GAMM.

Variance Term Estimation

For random intercept terms, by analogy with the way that mixed-effect model results are usually reported and light of the fact that we wish to control for any individual or day-to-day effects rather than performing hypothesis testing for them, it is possible to report the standard variance components associated with each term. They are shown below (in units of standard deviation). Note that since we used maximum-likelihood estimation, these estimates may be biased for finite sample size, but since variance term estimation is not the focus of inference (and smooth term hypothesis testing is) this seems like the right choice in our case.

```
vars <- invisible(gam.vcomp(HIF_model))

vars <- vars |>
  data.frame() |>
  mutate(
    Term = rownames(vars),
    Term = ifelse(Term == "scale", "scale (residuals)", Term)
```


Journal of Experimental Biology • Supplementary information

```
  ) |>
rename(`Standard Deviation` = std.dev,
       `CI (lower)` = lower,
       `CI (upper)` = upper
       ) |>
  select(Term,
         `Standard Deviation`,
         `CI (lower)`,
         `CI (upper)`) |>
  mutate(across(where(is.numeric), function(x) signif(x, 3))) |>
  gt()
```

```
vars
```

Term	Standard Deviation	CI (lower)	CI (upper)
s(exact)	4.79e-04	1.48e-04	0.00154
s(percentdailytotal)	2.42e-01	8.11e-04	72.20000
ti(exact,percentdailytotal)1	8.24e-05	7.27e-07	0.00934
ti(exact,percentdailytotal)2	1.38e-03	1.98e-06	0.96400
s(body_mass)	1.11e-07	0.00e+00	Inf
s(age)	2.69e-07	0.00e+00	Inf
s(pool_temp)	4.97e-06	0.00e+00	Inf
s(animal)	1.31e-01	7.24e-02	0.23600
s(day)	8.68e-02	4.50e-02	0.16700
scale (residuals)	2.00e-01	1.72e-01	0.23200

Table S 3: Variance components associated with each term in the GAMM.

Model prediction note

A way of visualizing the results of a GAM (or other regression model) is a prediction plot, where you select specific values at which to fix all the predictors other than `exact` time (age, proportion total calories, etc.) and then show predicted oxygen consumption according to the model – given those fixed values, and varying values of exact. Such a plot might be preferable to a partial plot because the y-axis is in terms of expected response variable values (in total, not a contribution to the whole from one predictor).

We used marginal means of "other" predictors; in this approach quantitative predictors are set to the mean value observed in the data, and for categorical predictors a weighted average across the observed values is used.

Random effects terms are not included, so that predictions are population-level (or, equivalently here, predictions for the typical dolphin on the typical day).

The idea is to try to show the expected response value for "an average observation" (in some sense) in the population, given the actual sample data and assuming the data is a reasonable sample of the population of interest. So, it may make the some sense as a set of predictions that can be overlaid on a plot of the data.

Marginal Means of Model Variables

We'd like to make model predictions with all predictors except for the time since feeding held constant at their mean values. What are those mean values?

Journal of Experimental Biology • Supplementary information

```
model_vars <- c('exact',
                'percentdailytotal',
                'body_mass',
                'pool_temp',
                'age',
                'sex')

tbl_summary(
    data = HIF_data,
    statistic = list(all_continuous() ~ "{mean} ({sd})",
                     all_categorical() ~ "{n} ({p}%)"),
    type = list(all_continuous() ~ "continuous",
                all_categorical() ~ "categorical",
                age ~ "continuous"),
    # type = assign_summary_type(variables = model_vars,
    #                            cat_threshold = 3,
    #                            value = NULL),
    include = all_of(model_vars)
  )
```

Characteristic	N = 133[1]
exact	52 (49)
percentdailytotal	0.23 (0.06)
body_mass	179 (22)
pool_temp	22.80 (1.91)
age	21 (14)
sex	
F	32 (24%)
M	101 (76%)

[1] Mean (SD); n (%)

Table S 4: Marginal means of data variables.

Predictions with uncertainty

To compute model-predicted oxygen consumption and total oxygen consumption (area under the curve), all with uncertainty, we can employ a resampling-based approach.

We use `mgcv::gam.mh()` to draw samples of model parameter values via Metropolis-Hastings sampling from the posterior.

Journal of Experimental Biology • Supplementary information

We obtain a linear-predictor matrix from the fitted model, which when post-multiplied by a vector of candidate parameter values, yields predictions from the fitted model for the chosen mean values of predictor variables (and varying values of `exact` time).

By generating these predictions for many parameter samples, we can obtain model predictions and derived quantities with uncertainty (here, percentile-based confidence intervals and standard errors are computed).

```
nsim <- 10000
set.seed(14)

# draws of parameter estimates from the posterior
actual_params <- matrix(coef(HIF_model), nrow = 1)
post_params <- gam.mh(HIF_model, ns = nsim*2, burn = 2000, thin = 2)$bs
# yields matrix with cols = model params and rows = sim #s

# get linear predictor matrix for "mm data"
mm_const <- HIF_data |>
  select(percentdailytotal,
         body_mass,
         age,
         pool_temp,
         is_male) |>
  summarise(across(everything(), function(x) mean(x)))

mm_df <- expand_grid(mm_const,
                     exact = seq(from = 0, by = 1, to = 133))

mm_lpmat <- predict(HIF_model,
        newdata = mm_df,
        type="lpmatrix",
        exclude = c("s(animal)", "s(day)"),
        newdata.guaranteed = TRUE)

# get predicted vo2 values (each col is one sim)
vo2_curve <- mm_lpmat %*% t(post_params) ## posterior curve samples
vo2_curve_actual <- mm_lpmat %*% t(actual_params)

# mean and 95% CI
vo2_predictions <- mm_df |>
  mutate(pred = apply(vo2_curve,
                      MARGIN = 1,
                      FUN = mean),
         ci_low = apply(vo2_curve,
                      MARGIN = 1,
                      FUN = function(x) quantile(x, 0.025)),
         ci_hi = apply(vo2_curve,
```


Journal of Experimental Biology • Supplementary information

```
                    MARGIN = 1,
                    FUN = function(x) quantile(x, 0.975)))
```

```
compute_auc <- function(predicted_smooth, x = mm_df$exact){
  AUC = cumtrapz(x = x,
                 y = predicted_smooth)
  total_AUC <- max(AUC)
  return(total_AUC)
}
```

```
vo2_totals <- apply(vo2_curve,
                    MARGIN = 2,
                    FUN = compute_auc)
vo2_totals_actual <- compute_auc(vo2_curve_actual)

# as percent, assuming BMR is constant
BMR_133 <- vo2_curve[mm_df$exact == 0,] * 133
BMR_daily <- BMR_133 * (24 * 60 / 133)
# 1051.2 # L O2 in one day

HIF_133 <- vo2_totals - BMR_133
daily_HIF <- HIF_133 / mm_const$percentdailytotal
daily_HIF_percent <- (daily_HIF / BMR_daily) * 100
```

Histogram of simulation results:

Journal of Experimental Biology • Supplementary information

```
gf_histogram(~vo2_totals) |>
  gf_labs(x = "Liters Oxygen Consumed (over 133 minutes)",
          y = "Number of Simulations")
```

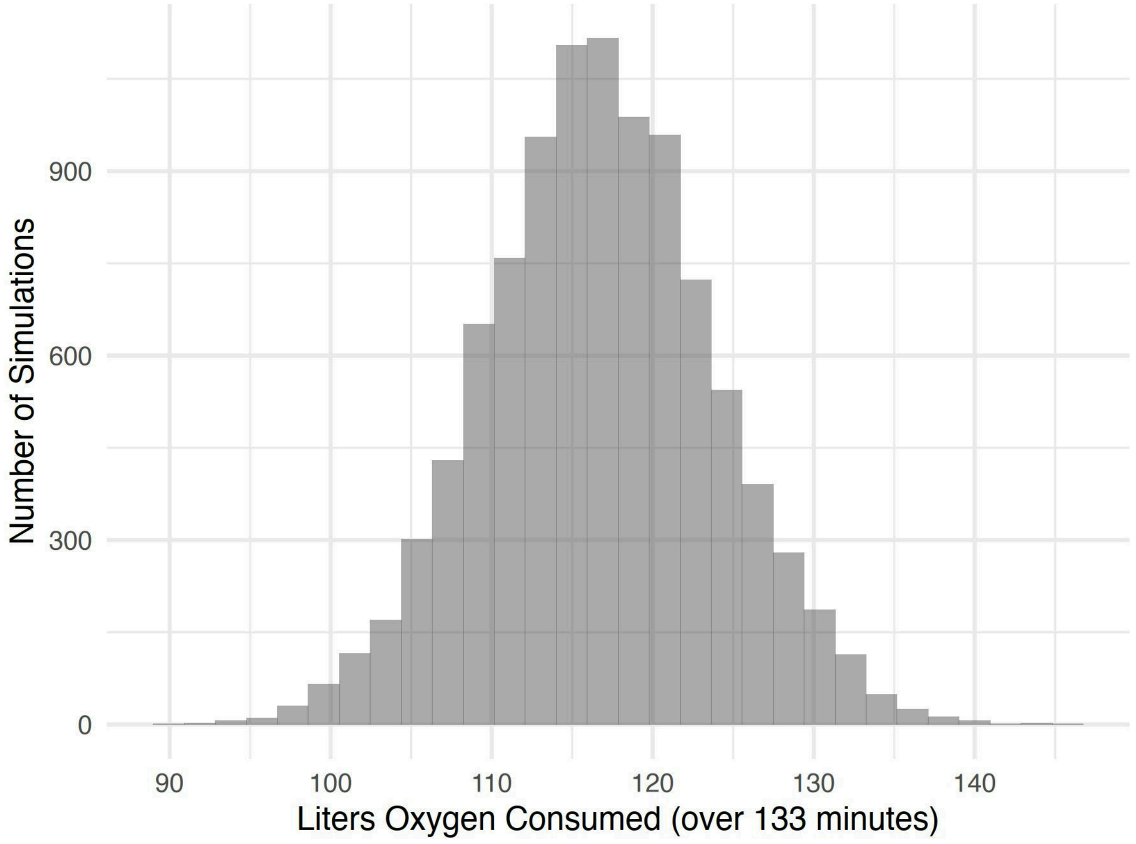

Fig. S5. Expected liters of oxygen consumed according to simulations.

Journal of Experimental Biology • Supplementary information

```
gf_histogram(~daily_HIF_percent) |>
  gf_labs(x = "HIF (percent of daily BMR)",
          y = "Number of Simulations")
```

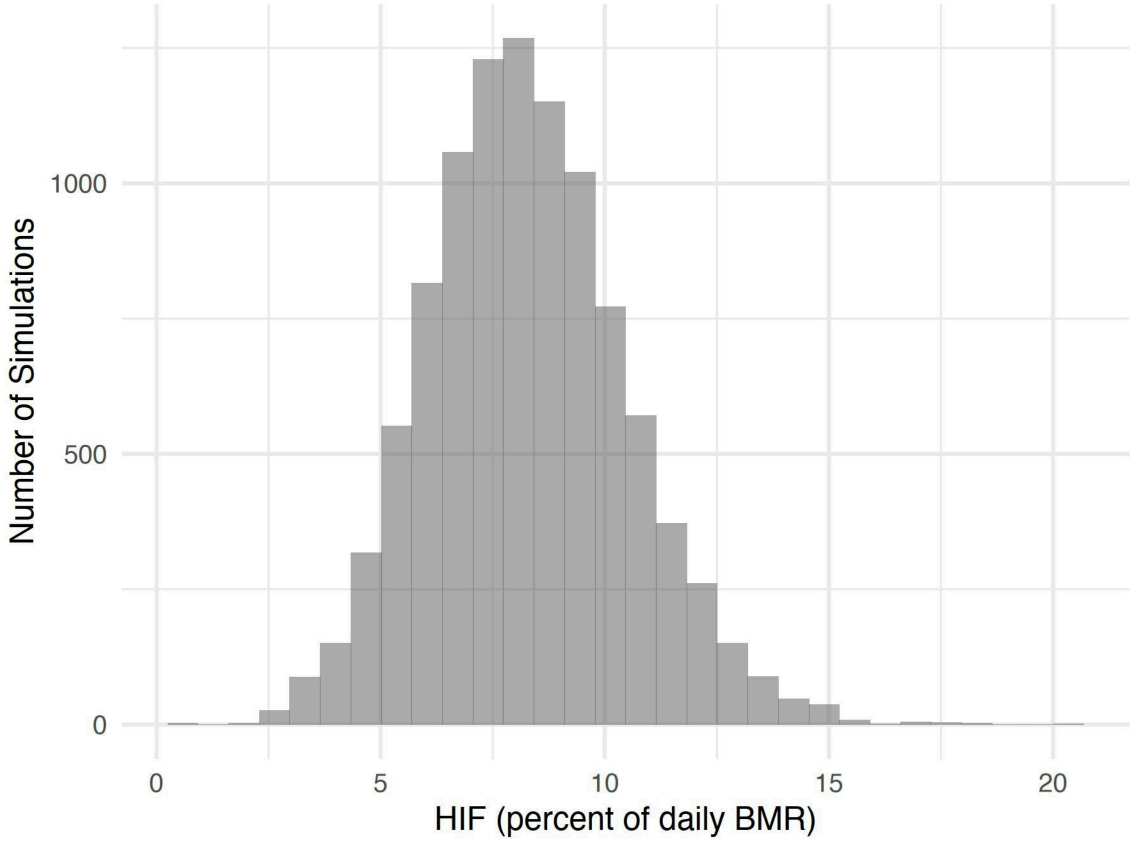

Fig. S6. Expected HIF as a percent of daily BMR, according to simulations.

Calculations of specific values of interest:

oxygen consumption over the 133 minutes:

```
vo2_mean <- round(mean(vo2_totals), digits = 2)
vo2_se <- round(sd(vo2_totals), digits = 2)

vo2_mean
```

```
[1] 116.7
```

```
vo2_se
```


Journal of Experimental Biology • Supplementary information

```
[1] 7.08
```

daily HIF total:

```
HIF_daily <- round(mean(daily_HIF), digits = 2)
HIF_daily_se <- round(sd(daily_HIF), digits = 2)
HIF_daily
```

```
[1] 85.73
```

```
HIF_daily_se
```

```
[1] 19.24
```

daily HIF as percent:

```
vo2_percent_mean <- round(mean(daily_HIF_percent), digits = 2)
vo2_percent_se <- round(sd(daily_HIF_percent), digits = 2)

vo2_percent_mean
```

```
[1] 8.26
```

```
vo2_percent_se
```

```
[1] 2.2
```

BMR over 24h:

```
BMR_daily_mean <- round(mean(BMR_daily), digits = 2)
BMR_daily_se <- round(sd(BMR_daily), digits = 2)
BMR_daily_mean
```

```
[1] 1051.27
```

```
BMR_daily_se
```

```
[1] 81.81
```

over 133 minutes, using the BMR at time 0:

```
# at time 0
mean(vo2_curve[mm_df$exact == 0,] * 133)
```

```
[1] 97.0969
```

```
sd(vo2_curve[mm_df$exact == 0,] * 133)
```

```
[1] 7.556422
```

The mean (standard deviation) of the simulated values is: 116.7 (7.08) liters.

The mean (standard deviation) of the simulated percentages is: 8.26 (2.2).

For additional reference if needed, the initial value of oxygen consumption at time 0 is: 0.73 (se 0.057), and so over the whole 133 minutes, 97.1 (se 7.56)

Data Plot with Model Fit

Define colors and shapes to use for plotting

```
kcal_colors <- c("#E41A1C", "#377EB8", "#4DAF4A", "#984EA3")
shape_vector <- c("Tt1" = 1, "Tt2" = 2, "Tt3" = 3, "Tt4" = 4,
                  "Tt5" = 5, "Tt6" = 6, "Tt7" = 7, "Tt8" = 8)
```


Journal of Experimental Biology • Supplementary information

```
oxygen_time_plot <- ggplot(HIF_data,
                           aes(x = exact, y = oxygen_cons)) +
  geom_point(aes(color = factor(kcal), shape = animal),
             size = 1,
             stroke = 1.2) +
  geom_line(data = vo2_predictions,
            aes(x = exact, y = pred),
            color = 'black',
            linewidth = 1) +
  geom_ribbon(data = vo2_predictions,
              aes(ymin = ci_low, ymax = ci_hi, x = exact),
              inherit.aes = FALSE,
              fill = 'grey44', alpha = 0.2) +
  labs(x = "Time (min)",
       y = "Oxygen Consumption (L / min)",
       color = "Energy Intake (kcal)",
       shape = "Animal ID") +
  scale_color_manual(values = kcal_colors) +
  scale_shape_manual(values = shape_vector) +
  theme_minimal() +
  theme(axis.text = element_text(size = 12),
        axis.title = element_text(size = 14),
        axis.line = element_line(linewidth = 1)) +
  scale_x_continuous(breaks = seq(0, 120, by = 20)) +
  scale_y_continuous(breaks = seq(0, 2, by = 0.2))

print(oxygen_time_plot)
```

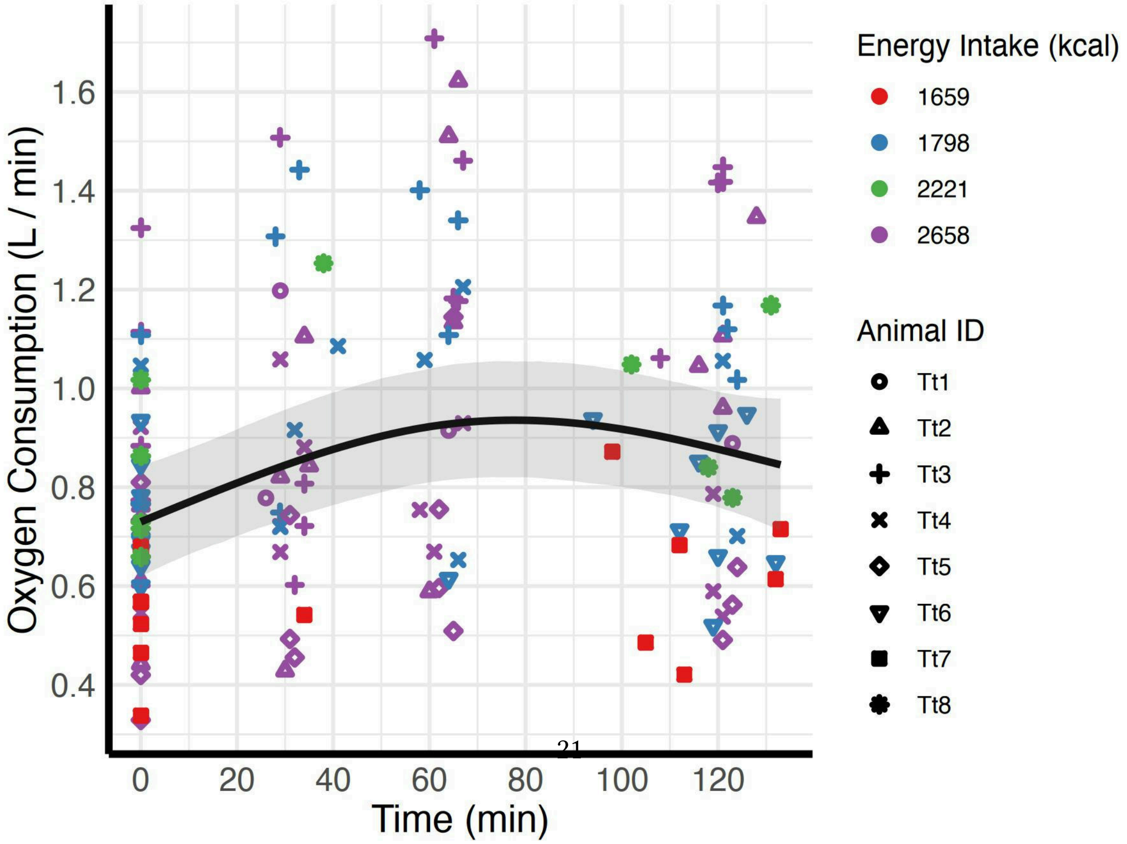

Fig. S7. This repeats the data-and-model figure from the main manuscript (to show code).

Journal of Experimental Biology • Supplementary information

```
ragg::agg_tiff("figures/figure-1.tiff",
               width = 6,
               height = 4.5,
               units = "in",
               res = 300)
oxygen_time_plot
dev.off()
```

```
pdf
  2
```

References

Hartig F (2024). *DHARMa: Residual Diagnostics for Hierarchical (Multi-Level / Mixed) Regression Models*. R package version 0.4.7, https://CRAN.R-project.org/package=DHARMa.
